# Charting the Unknown: Sex Differences in Spatial Exploration Across the Lifespan

**DOI:** 10.1007/s12110-025-09492-y

**Published:** 2025-05-29

**Authors:** Luming Zheng, Zehra Nur Genç, Valentin Baumann, Ineke van der Ham, Judith Schomaker

**Affiliations:** 1 Faculty of Social Sciences, Leiden University, Leiden, The Netherlands; 2 Department of Child and Adolescent Psychiatry and Psychotherapy, University of Magdeburg, Magdeburg, Germany; 3 Leiden Institute for Brain & Cognition, Leiden, The Netherlands

**Keywords:** Exploration, Sex differences, Lifespan, Navigation, Efficiency

## Abstract

Previous work has suggested that males and females adopt different exploration styles, with females often taking a more secure, or cautious exploration approach. However, exploration behavior is multi-faceted, and previous studies often investigated only specific aspects of it. As a result, sex differences in exploration remain vaguely defined, and it currently remains unclear when differences develop. In the current study, females (*n* = 206) and males (*n* = 218) aged 7–77 years explored a 3D virtual environment. First, we computed a series of exploration measures that are commonly used in the animal literature (including roaming entropy, distance traveled, shape of exploration and exploratory efficiency). Taking a fine-grained approach, based on a hierarchical cluster analysis we identified three distinct measures of exploration: (1) Exploratory activity; (2) Exploratory efficiency; (3) Shape of exploration. We investigated whether these distinct aspects capture individual differences– between sexes and across the lifespan– in exploration behavior. Males were found to explore more than females, as evidenced by higher exploratory activity and a more complex shape of exploration. Interestingly, however, females explored more efficiently than males. No interaction between age and sex was observed, suggesting that the effect remains stable across the lifespan. These findings suggest that even though women show more caution when exploring an unknown environment, they are more efficient in charting it. Our novel fine-grained exploratory behavior analyses further suggested that older individuals had a simpler exploration shape– as defined by lower sinuosity/tortuosity in their path– than younger individuals, while other exploration measures were not affected by age. The current novel findings thus suggest that specific characteristics of exploration behavior depend on individual differences, including sex and age. Previous work has suggested that exploration behavior is affected in several neuropsychiatric disorders (e.g., in schizophrenia), and our measures of exploration could potentially be used to characterize unique aspects of these disorders, and serve as cognitive markers.

**Supplementary Information:**

The online version contains supplementary material available at 10.1007/s12110-025-09492-y.

## Introduction

Exploration of novel territory is vital for securing resources and is a central characteristic of mammalian behavior. It involves weighing physical risks (e.g., fatigue, dehydration, potential attackers) and potential rewards in novel environments against exploiting known, but limited resources in a familiar environment. Typical findings in exploration studies suggest that females adopt a more cautious exploration style than males (Gagnon et al., 2016). According to the hunter-gatherer hypothesis (Silverman & Eals, 1992), such sex differences observed in navigation and exploration in modern-day humans can be led back to a division of labour in prehistoric hunter-gatherer communities. In these communities, men were presumed to be hunters who would walk long distances in uncharted territory, while females were presumed to be gatherers who would take care of offspring and/or gather resources mostly in familiar terrain (Hughes et al., 2014; Lombard & Kyriacou, 2020). This theory, however, has been challenged by archeological findings and a meta-analysis that suggests that women also adopted the role of hunters (~ 9.000 years ago; Haas et al., 2020). Although these sex differences in hunter-gatherer populations have been put forward to explain current-day differences in navigation skills between men and women it remains unclear how these role divisions are reflected in modern-day differences in exploration styles.

In line with the hunter-gatherer hypothesis, however, studies on spatial exploration have suggested that females adopt a more cautious exploration style than males. For example, one interpretation of the sex differences is that females show more harm avoidant behavior, than males in a 3D exploration task (Gagnon et al., 2016). Along a similar vein: Males have been reported to have a large range, while females have been reported to adopt a more cautious exploration style, for example revisiting previously visited landmarks more often and exploring a smaller area than males (Gagnon et al., 2016, 2018). In addition, females have also reported higher spatial anxiety than males (van der Ham et al., 2021). Related to exploration is navigation. Both depend on spatial orientation, but while exploration involves free movement without a clearly defined aim, navigation is goal-oriented. Findings on navigation are equivocal: Some studies suggest that males outperform females (e.g., Harris et al., 2019), while more fine-grained approaches suggest that males and females use different cues or may adopt different navigation styles (e.g., Sandstrom et al., 1998), but do not differ in terms of performance (also see: Spence et al., 2009). Taken together, there is inconclusive evidence that males and females use different approaches in navigation and exploration tasks, but the development and precise nature of these differences remains poorly defined.

Spatial orientation is a fundamental ability that develops gradually during childhood. A meta-analysis (Pullano & Foti, 2022) suggests that children already begin to orient themselves using coincident cues for navigation as early as 4.5 to 6 months of age. Newcombe and Ratliff (2007) suggested that between 18 and 24 months toddlers have developed spatial reorientation abilities, and that a critical starting for the development of spatial thought point is around 2 years of age, when spatial coding dependent on the hippocampus - i.e., place learning - emerges (Newcombe & Janellen Huttenlocher, 2000). Children aged 3 to 6 years demonstrate basic spatial coding abilities in tasks such as spatial perspective-taking and judgments of distance, enabling them to represent object locations and retrieve these locations based on reference points (allowing them to adopt both egocentric and allocentric frames of reference; Nardini et al., 2006). By age 6, children demonstrate the capacity to integrate geometric and featural information from the environment and utilize spatial language (e.g., “left” and “right”). During middle childhood (6 to 12 years old), children have developed the ability to integrate both proximal and distal cues, with their navigation skills approaching adult-like levels. This ability peaks in adolescence and declines in adulthood (van der Ham et al., 2020). Although there is no evidence to suggest the presence of gender differences in navigation abilities during childhood (van der Ham et al., 2020; Pullano & Foti, 2022), it remains unclear whether such differences emerge in adulthood or later in aging.

Navigation and exploration strategy differences could be due to innate (i.e., biological) or cultural factors. On one hand, studies on stereotypes suggest that priming a male stereotype can enhance womens’ performance on spatial tasks, diminishing sex differences (Ortner & Sieverding, 2008), and a large-scale study (*n* = 7150) suggested that males overestimate, while females underestimate their spatial navigation ability (van der Ham et al., 2020). Thus, spatial skills may be partly fluid and influenced by expectations or beliefs about one’s performance compared to others (Miola et al., 2023), and sex differences in spatial navigation have been reported to be smaller in younger children (e.g., < 13 years), than in older individuals, as evidenced by smaller effects sizes or no gender differences (Nazareth et al., 2019; van der Ham et al., 2020). These findings suggest that cultural factors (e.g., stereotype effects, self-perception biases) may play an important role in shaping sex differences in navigation ability across development, alongside potential biological influences. On the other hand, some studies already observe a male advantage in spatial transformation tasks in children as young as 4.5 years, even before the development of pervasive stereotypes (Banse et al., 2010; Levine et al., 1999). With increasing age, children’s mobility range expands, as caretakers permit a broader scope of exploration (Schug, 2015; Marzi et al., 2018). Developmental work has suggested that compared to female children, male children have significantly larger wayfinding ranges, a higher tendency to use orientation strategies, and lower levels of wayfinding anxiety (Schug, 2015). The controversy in the literature likely suggests that both biological and cultural factors are at play, and it is possible that due to influence of cultural effects beliefs, sex differences change across the lifespan.

Previous studies have already suggested that there is a shift from exploration towards exploitation across the lifespan (Lloyd et al., 2023). For example, in a reward search task children (7–11 years of age) explored more than adults but were also less effective in gathering rewards (Schulz et al., 2019). These findings suggest that children may be more on the exploration side of the exploration-exploitation trade-off. Also other types of exploration behavior have been reported to vary across the lifespan. In one study, reduced exploration behavior was observed in two foraging tasks in older (~ 70 years) versus younger (~ 24 years) individuals (Mata, Wilke, & Czienkowski, 2009; Mata et al., 2013). Similarly, in a food choice task older compared to younger participants chose fewer surprise items, suggesting a reduction in exploratory behavior with increasing age (but note, this study included relatively young participants between 18 and 30 years of age; Petzke & Schomaker, 2022). The respective impact of both biology and environment on exploration behavior between the sexes could be addressed by investigating the interaction between age and sex, but currently studies taking into account both of these factors are scarce.

Discrepant findings in spatial exploration and navigation studies may in part be due to the large differences in how exploration is operationalized between studies. For example, spatial exploration behavior can be observed in 3D naturalistic environments (e.g., Einhäuser et al., 2007; Prpic et al., 2019; Schomaker et al., 2014, 2022), in foraging tasks (Kalff et al., 2010; Lloyd et al., 2023), in visual search tasks (Wu et al., 2018; Bella-Fernández et al., 2022), and decision-making tasks (e.g., Mehlhorn et al., 2015; Apicella et al., 2017; Petzke & Schomaker, 2022). The few studies investigating exploration in 3D environments, typically focused on only a few aspects of exploration behavior (e.g., area covered or distance traveled). In the current study, we therefore aimed to take a more fine-grained approach, by taking into account a large number of exploration measures (Baumann, 2024; Baumann et al., 2025).

A lot of work has focused on target-oriented tasks, while tasks in natural environments are often absent or more loosely defined. Therefore, in the current study we aimed to investigate exploration behavior using *free exploration* of 3D spatial environments. This type of unconstrained free exploration has been described as undirected or intrinsically motivated behavior (Berlyne, 1960; Gottlieb et al., 2013), and novel environments have been suggested to specifically promote such behaviors (Düzel et al., 2010; Petzke & Schomaker, 2022). People frequently encounter real-world environments with broadly defined goals (e.g., there are numerous ways to prevent hunger, and it is up to an individual how to deal with this) and uncertain outcomes (Romer, 2010), while lab-based exploration tasks are often highly structured, and the goal is clearly defined. For example, specific items need to be foraged, or a well-defined target needs to be identified in a visual search task, and the behavior required to obtain that goal is more constrained. In this sense, free exploration tasks mimic real-world scenario’s where tasks can be more open. An important advantage of free exploration tasks furthermore is that they can capture a broader range of spontaneous exploration behavior, without imposing specific task demands.

In the current study, we therefore aimed to investigate specific characteristics of free exploration of a 3D environment in both males and females across the full lifespan (*n* = 424). In our experiment, males and females from a wide age-range (7–77 years) explored an unknown virtual environment (VE; a fantasy island with significant landmarks) for several minutes. Using a novel approach - relying on a hierarchical cluster analysis– we uncovered a nested structure of exploration measures (Baumann, 2024; Baumann et al., 2025), and identified three main clusters capturing disparate aspects of spatial exploration. On basis of the previously conducted hierarchical cluster analysis we calculated three compound measures (each including a series of more basic exploration measures): (1) Exploratory activity; (2) Complexity of the shape of exploration; and (3) Exploratory efficiency. Previous studies often focused on one or just a few types of exploration behavior. Our more fine-grained approach allowed us to investigate the development of individual differences in specific aspects of exploration behavior between sexes. We hypothesized that males would show more exploratory activity and potentially a more complex shape of exploratory behavior than females (e.g., Gagnon et al., 2016), and that sex differences develop during the lifespan. As our measure of exploratory efficiency captures a novel, less investigated aspect of exploration, we did not have a specific hypothesis with regard to sex differences.

## Methods

### Participants

This study was conducted as part of a Science Live program at the NEMO Science Center in Amsterdam. Data were gathered during the 2-week period, from 487 visitors aged 7-77who expressed their willingness to participate in our study. Prior to initiating the study, we gave all participants, or a participant’s parent in the case of minors, a written informed consent document containing information about the research’s purpose, procedures, potential risks, and benefits. Participants had the option to carry out the tasks either in Dutch or English.

From the original sample of 487 participants, 55 participants were not included in the main analyses, due to technical (*n* = 7; e.g., a task crash), organizational (*n* = 11; e.g., incorrect task order), administrative (*n* = 17; e.g., unintended reuse of a participant number) or other issues (*n* = 20; e.g., language issues). Ten participants were excluded, as they were identified as outliers in the main exploration analyses (|mean| > 3*SD; 3 outliers for cluster 2; 7 outliers for cluster 3). An additional three participants were excluded as they indicated “other” on the sex question. The final sample included 424 participants, including 206 females, and 218 males aged 7–77 years old (mean = 23.90; SD = 16.81). Age was treated as a continuous variable in the main analyses, but additionally an age group analysis was performed. See Supplementary Materials S1 for the number of male and female, younger children (7–11 years), adolescents (12–17 year), younger adults (18–44) and older adults (45–77 years) per age group.Participants explored either one of two VEs (VE 1 [green], VE 2 [pink]). Which VE participants explored first (the data that is reported here) was randomly assigned (105 females and 106 males explored VE1; 101 females and 112 males explored VE 2). We used two environments to counterbalance and control for potential environmental differences in our novelty manipulation, which was particularly relevant within the scope of the broader study (e.g., see Schomaker et al., 2022). Note that here we only report the results from the first exploration round, when both VEs were still novel.

The study’s procedures were aligned with COVID-19 guidelines and conducted in compliance with the Declaration of Helsinki (1964 and subsequent amendments). The research received approval from the Psychology Research Ethics committee (CEP) at Leiden University, the Netherlands (approval code: 2020-09-24-J.Schomaker-V2-2622).

### Stimuli and Apparatus

**Fig. 1:**
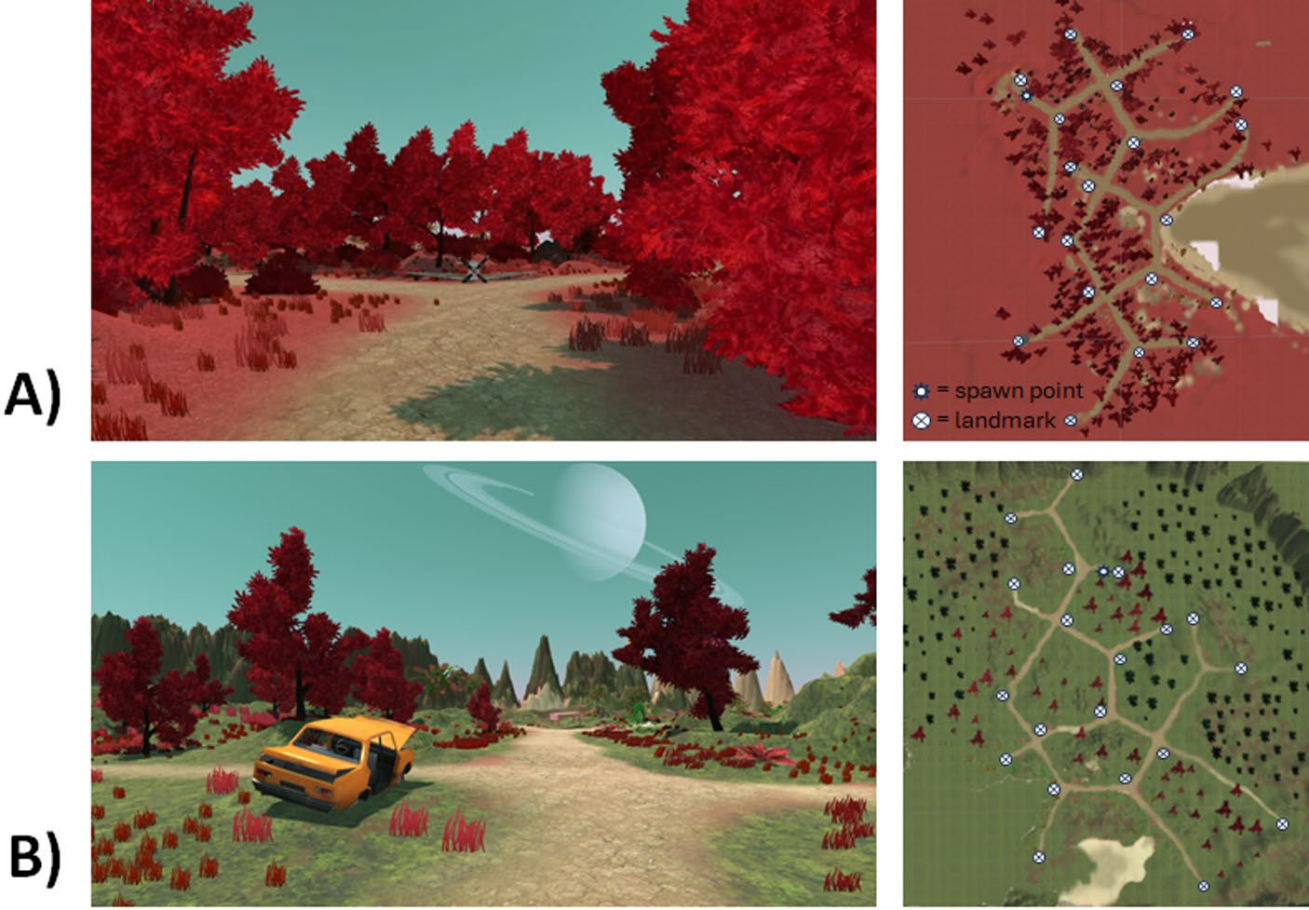
Virtual environments. The two virtual environments (VEs): (**A**) pink and (**B**) green, each including twenty landmarks (e.g., a plane and a car), from a first-person perspective (left) and a top view (right). In the current study exploration data of the first exploration round is included, which consisted of one of these VEs (counterbalanced between participants). The star indicates the location of the spawn point in each VE, and the circle markers indicate the location of the landmarks

The VEs (see Fig. 1) ran on laptops operating with Windows 10 and the two VEs that were used were created with Unity Version 2017.2.21f1 (Unity Technologies, 2017). The VEs were similar in terms of size, path length, and the number of intersections. In both VEs, participants had the option to move forward by using the W key on the keyboard and determine the direction of movement through the mouse. The VEs comprised fantasy islands featuring twenty unusual landmarks (such as a slot machine and a Halloween pumpkin) located at intersections or road endpoints (locations of the landmarks are shown in Fig. 1). Both VEs included both land and a body of water. Throughout the exploration process, the X, Y, and Z coordinates of the mobile agent were recorded at all time intervals, with a sampling rate of approximately 15 Hz. The landmark task and Novelty Seeking questionnaire (not reported here; see Schomaker et al., 2022) were developed using E-Prime 3.0 software (Psychology Software Tools, Pittsburgh, PA), while the word memory task (not reported here; see Schomaker et al., 2022)and ratings were carried out through Open Sesame 3.3.347. The main statistical analyses were performed using IBM SPSS Statistics 27. The Bayesian statistics were performed using JASP 0.13.10 (Love et al., 2019).

### Procedure

The current research was part of a larger study conducted at the NEMO Science Center in Amsterdam. To ensure social distancing ( > = 1.5 m), the experimenters utilized six laptops in two large testing rooms and wore masks and gloves throughout the study. Participants provided informed consent before participation. Throughout the experiment, the experimenter stayed with the participants in the testing room to monitor and initiate tasks and answer questions. In this paper we report the results from the exploration task. Results from other tasks are reported elsewhere (e.g., Schomaker et al., 2022; Ruitenberg et al., 2022).

The experiment consisted of two exploration phases. In the first familiarization phase, participants were given verbal scripted instructions on how to navigate in the presented VE, and they were asked to explore these VEs freely. Participants had 3 min in this phase to explore the VE and were instructed to make an effort to remain on the paths. Pressing the “W” key enabled participants to move forward, and the mouse was used for observing the surroundings and determining the direction of movement. Participants had the option to use the space bar for jumping; nevertheless, since jumping lacked a specific purpose, they couldn’t leap onto objects. Following the exploration, participants were prompted to use number keys to express their level of happiness on a scale from 1 (extremely unhappy) to 9 (extremely happy) and their arousal level from 1 (very calm) to 9 (very excited) on a visual analogue scale (VAS) with Self-Assessment Manikins. The evaluation was completed in less than one minute. Following the evaluation, the second exploration phase commenced. In this phase, participants were presented with another VE, which could be either the same (“familiar”) or different (“unfamiliar”) from the environment they had previously experienced. Note, in the current study we only analyze exploration data for the first round of exploration (i.e., the familiarization round), when the environments were still novel. In this stage, just like the initial phase, it lasted for three minutes. After completing this exploration, participants were instructed to again evaluate their levels of happiness and arousal level using the same two visual analogue scales (VAS) as they did in the first exploration.

After these ratings participants performed a word memory task (results not reported here; see Schomaker et al., 2022), which took around 3–4 min. After completion of the word memory test, participants performed a ± 3-minute visual-motor adaptation task (results not reported here; see Ruitenberg et al., 2022). After this phase, participants performed a landmark test aimed to evaluate participants’ memory for landmarks encountered during the second exploration phase (results not reported here; see Schomaker et al., 2022).

Finally, participants were asked to provide information about their ages, genders, and handedness. Afterwards, participants over the age of 17 then completed the 34-item Novelty Seeking scale of the Tridimensional Personality Questionnaire while younger individuals filled out a simplified and condensed version of the questionnaire consisting of 20 items (results reported in Schomaker et al., 2022). The survey inquiries persisted on the screen until participants supplied responses to each one (“X” = yes; “M” = no). Participants answered all questions within an average of 2–5 min. Following this, feedback was presented based on the overall Novelty Seeking score, which was further categorized into low, medium, and high scorers. These cut-off scores were exclusively employed for delivering feedback to the participants and were not utilized in any analyses. The participants completed the entire experimental procedure in ± 15–25 min.

### Exploration Measures

In the current study we used exploration measures as defined by Baumann (2024) and Baumann et al. (2025). See Table 1 for descriptions of the different measures we used. Prior to conducting the hierarchical cluster analysis, all exploration measures were standardized to eliminate differences in scale. The researchers performed hierarchical clustering on standardized indicators (see Table 1) using the R package ClustOfVar, employing squared pearson correlation coefficients as the similarity metric. Cluster numbers were determined by analyzing the dendrogram, aggregation height plots, and applying the kneedle algorithm. Applying this approach to two different datasets (the NEMO data also used in the current study and the SILCTON dataset) the results confirmed that the three exploration dimensions—exploratory activity, shape of exploration, and exploratory efficiency—were stable across different task environments and participant groups.

*Exploratory activity* measures the extent and breadth of exploration, reflecting the spatial coverage and movement variability of an individual in a novel environment. It indicates the overall level of exploratory engagement and whether the individual systematically explores the entire space. *Shape of exploration* characterizes the spatial structure and complexity of exploration trajectories, assessing the degree of path tortuosity. It distinguishes between more linear, efficient exploration and highly curved, complex paths, which may indicate a more random or less efficient exploration strategy. *Exploratory efficiency* evaluates the effectiveness of exploration by assessing the extent to which individuals revisit previously explored areas and optimize information acquisition. Higher efficiency indicates broader environmental coverage with minimal redundant paths, whereas lower efficiency is associated with frequent revisits and suboptimal spatial utilization.

**Table 1:** Exploration measures and clusters

Exploration measure	Inverted	Definition/Computation
*Cluster 1*: *Exploratory Activity*		
Path Length	No	Total length of the trajectory
Pausing	Yes	Time spent without movement
Area Covered	No	Area covered during exploration
Roaming Entropy	No	Distribution of the frequency of movement across the area
Landmark Visits	No	Number of landmarks visited
*Cluster 2*: *Shape of Exploration*		
Fractal Dimension	No	Tortuosity of the trajectory
Sinuosity	No	Sinuosity of the trajectory
*Cluster 3*: *Exploratory Efficiency*		
Landmark Revisits	Yes	Number of returns to landmarks
Revisiting	Yes	Average number of returns to already visited places
Turnarounds	Yes	Number of turns with an angle > 180 degrees
Flight Turnarounds	Yes	Number of turns with an angle > 180 degrees (flight scale)
Area Efficiency	No	Area covered/distance traveled
Landmark Efficiency	No	Landmarks visited/distance traveled

*Note: We inverted four measures– i.e.*,* pausing*,* revisiting*,* landmark revisits*,* and flight turnarounds– such that higher values reflect more exploration for all measures. More details can be found in* Baumann (2024*) and* Baumann et al. (2025)

### Statistical Analyses

First, we z-scored all exploration measures. On basis of a hierarchical cluster analysis (as reported here: Baumann, 2024; Baumann et al., 2025; also see Table 1), we defined three exploration measures: (1) Exploratory activity; (2) Exploratory efficiency; (3) Shape of the exploration. *Exploratory Activity* included path length, pausing (inverted), area covered, roaming entropy, and landmarks visited. *Exploratory Efficiency* included revisiting (inverted), landmark revisits (inverted), flight turnarounds (inverted), area efficiency and landmark efficiency, and *Shape of Exploration* included sinuosity and fractal dimension. Note that we inverted four measures– i.e., pausing, revisiting, landmark revisits and flight turnarounds– such that higher values reflect more exploration for all measures.

To investigate the effects of sex, and age on exploration behavior we ran a 2*3 mixed ANCOVA with sex (male; female) as between-subjects factor, exploration type (exploratory activity; exploratory efficiency; shape of the exploration) as a within-subjects factor, and age as a covariate. The same ANCOVA was ran including the factor VE (pink or green; see Fig. 1) to investigate whether exploratory behavior differed between the two VEs, but no difference was observed (*p* =.858), nor did including this factor influence the pattern of results, and we therefore left this factor out of the main ANCOVA. To further investigate age differences, we ran a mixed 2*3*4 ANOVA with sex (male; female) and age (younger children; adolescents; younger adults; older adults) as between-subjects factors and exploration type (exploratory activity; exploratory efficiency; shape of the exploration) as within-subjects factor. The results of this age group analysis are reported in the Supplementary Materials S1.

The interaction exploration type and sex was followed up with three post-hoc independent *t*-tests to compare males and females per exploration type. The interaction between age and exploration type was followed up with correlation analyses between age and exploration for each type of exploration. An alpha threshold of 0.05 was used for all statistical analyses. For the follow-up analyses, Bonferroni correction was applied to compensate for multiple comparisons. To further investigate a null effect of interest (i.e., the interaction between sex and age) we performed Bayesian statistics using JASP. The Bayes Factor was reported alongside the main ANOVA result.

Finally, we checked whether any differences in exploration behavior could be explained by subjective reports of mood or arousal. For this we compared the pre-experiment and post-exploration mood and arousal ratings for males and females with independent-samples t-tests.

## Results

**Fig. 2:**
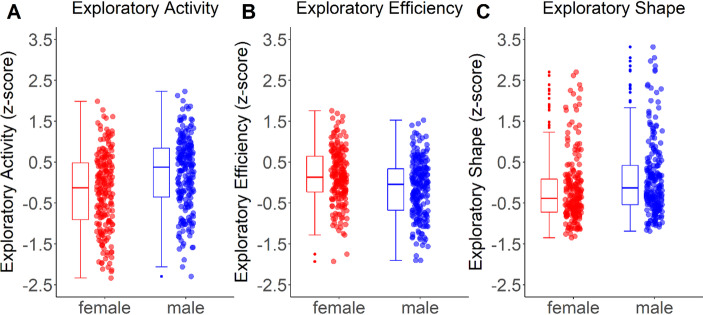
Mean z-scores per exploration type for both females and males. (**A**) Exploratory activity, (**B**) Exploratory efficiency, and (**C**) Exploratory shape

Figure 2 shows the mean z-scores per exploration type for males and females separately. The main 2*3 mixed ANCOVA revealed no main effect of exploration type, *F*(1.78,777.7) = 2.07, *p* =.126, *η*^2^ = 0.005 (Greenhouse-Geisser corrected). Males scored higher on the different exploration measures than females, as indicated by a main effect of sex, *F*(1,420) = 8.26, *p =*.004, *η*^2^ = 0.019. Age influenced exploration behavior, with older individuals scoring lower on the exploration measures than younger individuals, *F*(1,420) = 8.26, *p* =.004, *η*^2^ = 0.019. Furthermore, exploration type and age, interacted, *F*(2,840) = 4.46, *p* =.012, *η*^2^ = 0.012. No interaction was found for the interaction between sex and age, *F*(1, 420) = 0.006, *p* =.938, *η*^2^ < 0.001. As we were interested to test whether the effects of sex and age were additive, we also performed Bayesian statistics. Using Bayesian analyses we compared the model including all main effects (exploration type; sex; age) and all interactions (i.e., the full model) to the model with all main effects (exploration type; sex; age) and interactions, but no sex*age interaction. The Bayesian analysis suggested strong evidence against a sex*age interaction, BF_01_ = 9.90, suggesting that the model without this interaction is approximately 10 times more likely than the model including the interaction sex and age, further suggesting the effects to be additive. Exploration type and sex also interacted, *F*(2,840) = 7.40, *p* <.001, *η*^2^ = 0.017. The three-way interaction sex*age*exploration type was not significant (*p* =.276).

**Fig. 3:**
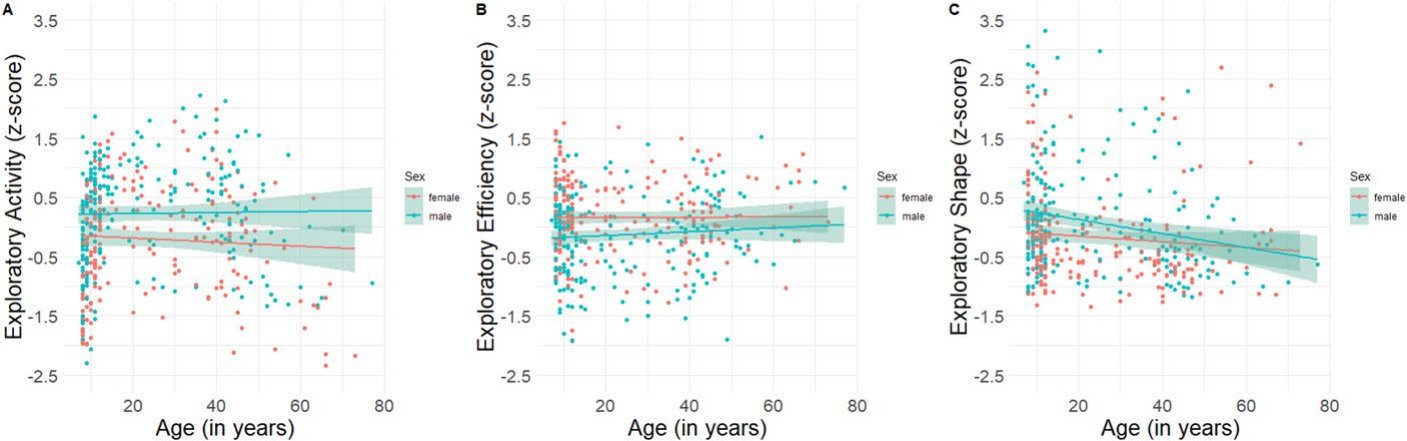
The relationship between age and the type of the exploratory behavior. The relationship between age and (**A**) Exploratory activity; (**B**) Exploratory efficiency and (**C**) Shape of exploration for both males and females. Age only correlated with the shape of exploration, which includes measures of sinuosity and fractal dimension. The negative correlation in panel C suggests that younger individuals showed a more complex shape of exploration than older individuals. The results suggested that the effects of age and sex were additive for exploratory activity and exploratory efficiency, as also evidenced by the mostly parallel lines. However, for the shape cluster we observed an age*sex interaction (also see Supplmentary Materials S1)

Figure 3 shows the relationship between age and exploration behavior per exploration type per sex (male; female). The interaction between exploration type and age was followed up with correlational analyses between age and exploration behavior for each type of exploration. Age did not correlate with exploratory activity (*p* =.546) or exploratory efficiency (*p* =.289), however, exploration behavior was less complex in older than younger individuals, *r*(424) = − 0.176, *p* <.001 (surviving Bonferroni-correction).

The interaction between exploration type and sex was further investigated with three independent post-hoc *t*-tests comparing males and females per exploration type. Males showed more exploratory activity than females, *t*(422) = 4.90, *p* <.001, and a more complex exploratory shape, *t*(422) = 2.96, *p* =.003. However, females explored more efficiently than males, *t*(422) = -4.49, *p* <.001. All comparisons survived Bonferroni correction.

Finally, males and females reported similar mood and arousal levels before (*p* =.588 and *p* =.939 respectively) and after exploration (*p* =.405 and *p* =.670 respectively). This suggests that mood or arousal did likely not significantly impact the sex differences observed in the other analyses.

## Discussion

In the current study, males and females across the lifespan (age 7–77 years) freely explored a VE. We used a novel, fine-grained analysis to characterize different aspects of exploration behavior allowing us to investigate specific sex differences in exploration behavior across the lifespan. Males showed more exploratory activity and a more complex shape of exploration than females, but females were more efficient in their exploration. In particular, females showed higher exploratory efficiency. No interaction between age and sex was observed, suggesting that the effect remained stable across the lifespan. In addition, it was found that younger individuals scored higher on exploration than older individuals. In particular, older individuals showed a simpler shape in their exploration behavior than younger individuals.

Our finding that males showed higher exploratory activity than females is in line with findings that females may adopt a more cautious exploration style than males (Gagnon et al., 2016) and modulated by sex hormones and cortisol ratios men exhibit more risk-taking behavior than females (Barel et al., 2017). For example, our exploratory activity cluster included roaming entropy, and our findings may suggest that females stayed on the paths more than males (as exploratory activity was generally lower in females than males). Previous work has suggested that males may adopt a more efficient navigation strategy, as evidenced by them taking more shortcuts and reaching a goal destination faster than females (Boone et al., 2018). For the exploratory efficiency cluster measures related to landmark revisiting, revisiting of locations, and (flight) turnarounds were reversed, such that a higher score reflects more efficient exploration. Additional measures in this cluster included area and landmark efficiency. Our findings for this cluster suggested that females explored more efficienty than males.

This finding contrasts earlier work that males adopt a more efficient navigation style (Gagnon et al., 2016). In that study, females paused longer and revisited landmarks more often than males (Gagnon et al., 2016). This difference may be explained by the differences in task instruction between the studies. Gagnon et al. (2016) used a navigation task in which participants were instructed to find five landmarks as quickly as possible, and to remember their location, as they would be asked to later point towards the location of the landmarks from a starting point. Potentially, females adopted a more cautious strategy due to risk aversity (e.g., fear of not remembering the correct landmarks for the pointing task) in the Gagnon et al. (2016) study, while our free exploration study did not guide exploration behavior in this way.

In contrast, females were found to explore more efficiently than males. In our exploratory efficiency cluster, higher efficiency reflects broader environmental coverage with minimal redundant paths, whereas lower efficiency is associated with frequent revisits and suboptimal spatial utilization. This sex difference in exploratory efficiency may be explained by females adopting a more landmark-based exploration strategy than males, eventhough our task involved free exploration (Sandstrom et al., 1998; MacFadden et al., 2003). Along a similar vein, females have been reported to rely more heavily on proximal rather than distal landmarks (Chamizo et al., 2011; Dahmani et al., 2023). A strategy based on local landmarks could potentially explain our current findings of higher exploratory efficiency in females, as keeping landmarks close would reduce the required distance traveled. Several models aimed at solving the traveling salesman problem (i.e., a classic optimization problem in which the goal is to find the shortest route to a set of given locations), use similar solutions of keeping targets close (e.g., using proximal optimization policies; Ryu, 2019; Le et al., 2021), suggesting that keeping landmarks close is part of an efficient navigation strategy. Note, however, that our efficiency cluster also included other types of measures, not specifically linked to landmark proximity or efficiency (e.g., revisiting and turnarounds), which could potentially explain the observed sex differences for this cluser. For example, it is possible that females revisited fewer places, or made fewer turnarounds, due to lower exploratory activity (e.g., a lower distance traveled).

Although males and females may use different information from landmarks, sex differences are often not observed when a near/local landmark-based strategy is optimal (Chamizo et al., 2011, 2014). This suggests that females may find target locations as effectively as men when they rely on allocentric information, such as landmarks (e.g., as in this study in rats: Chamizo et al., 2014). Keeping landmarks close could be part of a strategy to gather local information in novel territory. In line with this are findings in mice that suggested that males explore more, but females tend to learn better when they explore (Chen et al., 2021). Female focus on landmarks has already been reported in a study that suggested that males and females scan maps similarly, but that females use landmarks and direction pointers (i.e., left/right) more often than males to describe routes, while males more often refer to cardinal directions (i.e., north/south/east/west; MacFadden et al., 2003).

This is one of the first studies addressing individual differences in exploration styles, addressing sex differences across the lifespan. If development of exploration styles would follow a similar pattern as the development of stereotypes (e.g., Bradbard et al., 1986), you would expect to see that sex differences would be larger with increasing age. In our main analyses, however, no interaction between age and sex was found, as was visible in the mostly parallel linear fits for males and females for the different exploration types (Fig. 3) and this was further supported by strong evidence for the null hypothesis, supporting the notion that the effects of age and sex are additive. These findings suggest that differences observed between males and females are similar across the lifespan or are already developed in children younger than our sample (i.e., < 7 years of age). Our age group analyses allowed us to zoom into these effects per exploration type. Here we confirmed that age and sex did not interact for the exploratory activity and exploratory efficiency clusters, however, for the shape cluster an interaction was found (also see Fig. 3). Follow-up tests suggested that for adolescents and younger adults, males showed a more complex shape of exploration, while no differences were observed for younger children and older adults. Although some work investigating exploration behavior in younger individuals already exists (e.g., Farran et al., 2022), future work using a more fine-grained exploration analysis, as put forward here, is needed to provide a more complete picture of the development of sex differences in exploration behavior. In addition, future studies could include direct measures of stereotypes to address the role of age in stereotype development and its potential effect on exploration behavior.

We did observe differences in exploration behavior across the lifespan: Our main analyses suggested that older individuals explored less than younger individuals. Our age group analyses (see S1) zoomed into this age effect and revealed that adolescents explored more than younger children, and younger adults explored more than older adults, which is in line with previous work that suggests that navigation ability peaks in adolescence and goes down during adulthood (van der Ham et al., 2020). Specifically, the complexity of the shape of exploration was reduced in older individuals, as evidenced by lower sinuosity/tortuosity in the path they travelled. These findings are in line with previous work that reported age-related decline in for example visual exploration (Kaneko et al., 2021) and the novelty seeking personality trait (Petzke & Schomaker, 2022; Shan et al., 2023). It has previously been suggested that humans undergo a transition from exploratory strategies in adolescence to exploitative strategies in adulthood (Lloyd et al., 2023). Our fine-grained analysis of spatial exploration behavior suggested that the decline may be specific for certain aspects of exploration– namely the complexity of the path– while other measures, including exploratory activity and exploratory efficiency, remained unaffected. It is possible, however, that older participants found it more difficult to use the controls in our task, and showed less complexity in their exploration behavior as a result. Future studies could potentially address this by taking gaming experience into account, or by quantifying or training agility in a separate task. A potential limitation of our study is that we did not control for computer literacy and /or video game experience. Although our task made use of straightforward controls (i.e., a single key to move forward and the mouse to determine direction), older participants may have found the controls more challenging which could have influenced their exploration behavior. Future studies could address this by further assessing these factors or incorporating a separate task to evaluate agility.

This typical change from exploration towards exploitation across the lifespan, has been suggested to be disrupted in several mental health disorders (Lloyd et al., 2023). For example, exploration behavior is overly represented or reduced in addiction (Mitchell et al., 2016; Wingo et al., 2016; Zhou et al., 2019), depression (Trimmer et al., 2015), and certain anxiety disorders, such as agoraphobia (Kallai et al., 2007). A more fine-grained understanding of exploration behavior thus is relevant for our understanding and the treatment of neuropsychiatric disorders in which exploration behavior is implicated (Minassian et al., 2010, 2011; Perry et al., 2010). Specific measures of exploration (e.g., exploratory activity, shape of the exploration, or exploratory efficiency) could potentially be used as ecologically valid behavioral markers in these illnesses.

In conclusion, our novel exploratory behavior analyses suggest that even though women may show more caution when exploring an unknown environment than men, as evidenced by lower exploratory activity and lower complexity of the shape of exploration, they are more efficient in their exploration behavior. Our analyses further suggested that older individuals had a simpler exploration shape than younger individuals, while other exploration measures were not affected by age. Interestingly, age and sex did not interact, which may suggest that sex differences in exploration strategies remain mostly stable across the lifespan (Reio & Choi, 2004). The current novel findings thus suggest that specific characteristics of exploration behavior are influenced by individual differences, including sex and age, suggesting that taking into account these differences in exploratory styles is relevant when interpreting and targeting exploratory behavior in clinical populations.

## Electronic supplementary material

Below is the link to the electronic supplementary material.

Supplementary Material 1

## Data Availability

All human exploration data log files of the data reported are stored here: http://zenodo.org/records/12522513. Scripts for data preparation and analysis, including the hierarchical cluster analysis on which the clusters used in this article were based on, can be found here: https://github.com/valentinbaumann/explorationMeasures.
