# Supplementary material for: Charting the Unknown: Sex Differences in Spatial Exploration Across the Lifespan
Source: Hum Nat. 2025 May 29;36(2):219–37. doi: 10.1007/s12110-025-09492-y (PMC12417250; doi:10.1007/s12110-025-09492-y)
Supplement: Supplementary file 1 — Supplementary Material 1 [file 12110_2025_9492_MOESM1_ESM.docx]

**Supplementary materials**

**S1. Age group analyses**

To further investigate the effects of age on exploration behavior, we ran an additional analysis with age group as a between-subjects factor. We ran a mixed 2*3*4 ANOVA with sex (male; female) and age (younger children; adolescents; younger adults; older adults; see Table S1 for specific age ranges and numbers per group) as between-subjects factors and exploration type (exploratory activity; exploratory efficiency; shape of the exploration) as within-subjects factor. Note that we used the same groups as in Schomaker et al., (2022; this study is based on the same sample) for between-study consistency.

There was a main effect of sex, with higher exploration scores for males than females, *F*(1, 416) =15.61, *p <* .001. In addition, there was a main effect of age group, *F*(3, 416) = 9.83, *p* < .001, *η*^2^ = .066. A Tukey HSD test to further investigate this effect of age group showed that adolescents explored more than younger children, mean difference = 0.18, standard error (SE) = 0.05, *p* = .003. Adolescents and younger adults did not differ, mean difference = 0.11, SE = 0.05, *p* = .148. However, younger adults explored more than older adults, mean difference = 0.20, SE = .05, *p* < .001.

No main effect of cluster was observed, *F*(2, 832) = 0.36, *p* = .698, *η*^2^ = .001. Cluster and sex interacted, *F*(2, 832) = 15.59, *p* < .001, *η*^2^ = .036, as well as cluster and age group, *F*(2, 832) = 13.53, *p* < .001, *η*^2^ = .089. Sex and age group did not interact, *F*(3, 416) = 1.07, *p* = .362, *η*^2^ = .008, however, a three-way interaction between sex, age group and cluster was observed, *F*(6, 832) = 2.21, *p* = .041, *η*^2^ = .016. These interactions were followed up with 2*4 ANOVAs per cluster with sex (male; female) and age (younger children; adolescents; younger adults; older adults) as between-subject variables.

For the cluster exploratory activity, a main effect of sex was found, with males exhibiting more exploratory activity than females, *F*(1, 416) = 25.23, *p* < .001. In addition, a main effect of age group was found, *F*(2, 416) = 26.66, *p* < .001. A Tukey HSD suggested that adolescents showed more exploratory activity than younger children, mean difference = 0.74, standard error (SE) = 0.12, *p* < .001, while adolescents and younger adults did not differ, mean difference = 0.13, standard error (SE) = 0.13, *p* = .748, while younger adults showed more exploratory activity than older adults, mean difference = 0.73, standard error (SE) = 0.13, *p* < .001. No interaction between sex and age group was found, *F*(3, 416) = 0.71, *p* = .548.

For the cluster exploratory efficiency, a main effect of sex was found, but with females exhibiting more exploratory efficiency than males, *F*(1, 416) = 14.88, *p* < .001. In addition, a main effect of age group was found, *F*(2, 416) = 5.11, *p* = .002. A Tukey HSD suggested that younger children showed more exploratory efficiency than adolescents, mean difference = 0.30, standard error (SE) = 0.10, *p* = .015, while adolescents and younger adults did not differ, mean difference = 0.17, standard error (SE) = 0.10, *p* = .324. Nor did younger and older adults differ in terms of exploratory efficiency, mean difference = 0.23, standard error (SE) = 0.10, *p* = .121. No sex and age group interaction was found for exploratory efficiency, *F*(3, 416) = 0.56, *p* = .640.

For the shape cluster, a main effect of sex was found, with males exhibiting a more complex shape in exploratory behavior than females, *F*(1, 416) = 7.40, *p* = .007. In addition, a main effect of age group was found, *F*(2, 416) = 4.96, *p* = .002. A Tukey HSD suggested that younger children and adolescents did not differ in terms of the shape of exploration, mean difference = 0.09, standard error (SE) = 0.13, *p* = .914. Adolescents showed a more complex shape of exploration than younger adults, mean difference = 0.37, standard error (SE) = 0.13, *p* = .029, while younger and older adults did not differ, mean difference = 0.09, standard error (SE) = 0.14, *p* = .905. An interaction between age group and sex was found, *F*(3, 416) = 3.97, *p* = .008. This was followed up with a univariate ANOVA per age group. For younger children no sex effect was found, *F*(1, 159) = 0.08, *p* = .778. For adolescents children, males showed a more complex shape of exploration than females, *F*(1, 62) = 10.29, *p* = .002. Similarly, for younger adults, males exhibited a more complex shape of exploration than female participants, *F*(1, 136) = 10.84, *p* = .001. For older adults, no effect of sex was observed, *F*(1, 59) = 0.62, *p* = .433.

Table S1. Participants

| **Sex** | **Age group** | ***n*** |
| --- | --- | --- |
| *female* |  |  |
|  | Younger children | 72 |
|  | Adolescents | 31 |
|  | Younger adults | 74 |
|  | Older adults | 29 |
| *Male* |  |  |
|  | Younger children | 89 |
|  | Adolescents | 33 |
|  | Younger adults | 64 |
|  | Older adults | 32 |
| *Total* |  | *424* |

*Note, younger children were aged 7-11 years, adolescents 12-17 years, younger adults 18-44 years, and older adults 45-77 years.*
